# Quality of care after a horizontal merger between two large academic hospitals

**DOI:** 10.1016/j.heliyon.2024.e38311

**Published:** 2024-09-29

**Authors:** Ilse J.A. Wissink, Michiel Schinkel, Hessel Peters-Sengers, Simon A. Jones, Alexander P.J. Vlaar, Karen J. Kruijthof, W. Joost Wiersinga

**Affiliations:** a Department of Medicine, Division of Infectious Diseases, Amsterdam UMC, University of Amsterdam, Amsterdam, the Netherlands; b Center for Experimental and Molecular Medicine (CEMM), Amsterdam UMC, location AMC, University of Amsterdam, Amsterdam, the Netherlands; c Department of Intensive Care, Amsterdam UMC, University of Amsterdam, Amsterdam, the Netherlands; d Epidemiology and Data Science, Amsterdam UMC, Vrije Universiteit Amsterdam, Amsterdam, the Netherlands; e Centre for Health and Delivery Science, NYU Grossman School of Medicine, New York, USA; f Amsterdam UMC, Vrije Universiteit Amsterdam, De Boelelaan 1117, Amsterdam, the Netherlands

## Abstract

**Background:**

Hospital mergers remain common, but their influence on healthcare quality varies. Data on effects of European hospital mergers are ill defined, and academic hospitals in particular. This case study assesses early quality of care changes in two formerly competing Dutch academic hospitals that merged on June 6, 2018.

**Methods:**

Statistical process control and interrupted time series analysis were performed. All adult, non-psychiatric patients, admitted between 01-03–2016 and 01-10-2022 were eligible for analysis. Primary outcome measure was all cause in-hospital mortality (or hospice), secondary outcomes were unplanned 30-day readmissions to same hospital, length of stay, and patients’ hospital rating. Data were obtained from electronic health records, and patient experience surveys.

**Findings:**

The mean (SD) age of the 573 813 included patients was 54·3 (18·9) years. The minority was female (277 817, 48·4 %), and most admissions were acute (308 597, 53·8 %). No merger related change in mortality was found in the first 20 months post-merger (limited to the pre-Covid-19 era). For this same period, the 30-day readmission incidence changed to a downward slope post-merger, and the length of stay shortened (immediate level-change −3·796 % (95 % CI, −5·776 % to −1·816 %) and trend-change −0·150 % per month (95 % CI, −0·307 % to 0·007 %)). Patients’ hospital ratings seemed to improve post-merger.

**Interpretation:**

In this quality improvement study, a full- and gradual post-merger integration strategy for a Dutch academic hospital merger was not associated with changes in in-hospital mortality, and yielded slight improved results for secondary quality of care outcomes.

## 1 Introduction

Western healthcare markets are characterized by ongoing merger and acquisition (M&A) activity [[1], [2], [3]]. In the Netherlands alone, 113 hospital M&A's have taken place since 1978 [4,5]. Generally, M&A's are associated with increased prices as negotiated by health insurers, while effects on the quality of care (QoC) vary [1,6,7].

On the one hand, M&A's can result in clinical advancements through increased service volumes, (sub)specialization, and integration of infrastructure, facilities, staff, and clinical know-how [[8], [9], [10], [11]]. On the other hand, major organizational changes can distract attention from primary care processes, and insufficient remaining competition in healthcare markets may lead to reduced incentives for quality improvement [6,8,12,13]. The reported QoC outcomes in prior studies are often inconsistent [1,[6], [7], [8],[14], [15], [16]]. To illustrate, a study among 246 acquired and 1986 non-acquired short-term acute care hospitals in the US reported no significant changes in 30-day mortality four years post-acquisition [1], while another study among 172 merged and 266 non-merged rural hospitals in the US found decreased mortality rates within the first five years for patients with acute myocardial infarction, heart failure, stroke, and pneumonia [15].

Heterogeneity in clinical outcomes may be attributed to major variations between hospital M&A deals. While they are often studied as a uniform intervention, the diversity between M&A's in terms of merger-objectives, integration strategies and hospital- and market characteristics is substantial. Some studies have started to relate M&A characteristics to clinical outcomes, such as geographic location, the deployed post-merger integration strategy, and involvement of private equity [2,6,15,17,18].

This study assessed QoC effects after a hospital merger in the Netherlands. M&A characteristics that may influence post-merger QoC in this case include academic hospital status, the healthcare market, and the applied integration strategy and speed. M&A's in academic hospitals that yield increased service volumes through concentration may benefit the QoC, since higher volumes of typical academic healthcare services have been associated with improved patient outcomes [11,19]. Limited studies in academic hospital M&A's have cautiously reported some positive outcomes [6,[20], [21], [22], [23]]. Paradoxically, however, other studies have reported a negative association between concentration in healthcare markets and QoC [13,24]. Furthermore, the present case deploys a full-integration strategy, an approach that has been associated with improved QoC in earlier research [6]. Studies outside the healthcare sector also found deeper integration to be associated with improved post-acquisition company performance [25,26]. Finally, the merger under review deploys a long-term integration strategy. Given the complexity and ambiguity of the impact of integration speed [25], it is important to evaluate the effects of such a strategy on the QoC. Most evidence on post-merger QoC originates from the United States, whereas most healthcare markets in the European Union exhibit substantial differences such as mandatory health coverage for all citizens, and hospitals being predominantly non-profit. Hence, generalizability may be limited.

This quality improvement study assessed the QoC effects of a Dutch horizontal merger between two formerly competing academic hospitals that deploy a full and long-term integration strategy. The insights contribute to knowledge on characteristics that potentially impact clinical outcomes after hospital M&A's [2].

## 2 Methods

**Study setting and merger process** Located at 12 km from each other the Free University Medical Center (VUmc) and Academic Medical Center (AMC) used to be competitors in the delivery of healthcare. Both were urban, multispecialty, academic hospitals, with affiliations to different universities. In preparation for a merger, the hospitals adopted the same Electronic Health Record (EHR) system, operational in both hospitals since 2016. On June 6, 2018 the hospitals merged, a process initiated with the consolidation of the executive boards and the rebranding as Amsterdam University Medical Centres (Amsterdam UMC). One of the main objectives for the horizontal merger was to further improve the QoC through concentration of academic health services, with the ambition to ‘offer patients the very best that modern medicine can possibly achieve’. It was deemed financially infeasible to construct one new hospital, leading to the decision to consolidate healthcare services over the two existing buildings. A full-integration strategy was developed to allocate one center to trauma and non-elective care, and the other to elective, chronic and oncological healthcare. This demanded a high degree of integration depth, in which practically all departments had to integrate with their counterparts. Due to merger related renovations, limited space and ongoing patient care, a gradual long-term integration strategy was outlined in which clusters of departments harmonize and concentrate successively in so called merger ‘waves’ (eFig. 1). Patients are moved from one location to the other if their admission falls within the merger wave of the admitting specialty. While the most significant repositioning of healthcare services has been established, the harmonization and integration practices are expected to continue until at least 2025. Details on the hospitals' merger process (eFig. 1) and the Dutch healthcare market and regulated competition (**eText 1**) can be found in the supplementary material.

### 2.1 Study design and data sources

The primary outcome measure was all-cause in-hospital mortality or discharge to hospice. Secondary outcome measures were unplanned-30-day-same-hospital-readmissions, length of hospital stay, and patients' hospital rating. The source for these data were the hospitals' EHR's, and they were chosen because of their high registration reliability, and due to their recognition as parameters of QoC [1,6,15,27,28]. Patients' hospital rating was assessed using the Consumer Quality Index (CQI), conducted in Dutch academic hospitals between 2013 and 2019, and the Patient Experience Monitor (PEM), conducted in Dutch academic hospitals from 2019 up until now [29,30]. Approximately 20 months after the hospitals merged, the Covid-19 pandemic broke out (the first SARS-CoV-2 infected patient in Amsterdam UMC was registered in February 2020). Successive national Covid-19 waves are depicted in the charts, as these could have an influence on the study outcome parameters [31].

### 2.2 Study cohort

All adult non-psychiatric patients, pre-merger admitted to the former AMC and VUmc hospitals and post-merger to Amsterdam UMC, were included in this study. Presentations at the emergency department were registered as being admissions. Data were collected from March 1, 2016 (the time point when Epic was implemented in both hospitals as the EHR system) until October 1, 2022. The official date of the merger was June 6, 2018, which marked the merger of the executive boards of both hospitals. For the main analysis, data were used up until the admission of the first Covid-19 patient. In the subsequent period, multiple healthcare services were scaled down which significantly changed the healthcare landscape.

### 2.3 Statistical analysis

Descriptive statistics were used to describe the pre- and post-intervention patient cohorts. For the post-merger period two cohorts are described, the first up until the admission of the first patient with Covid-19, and the second from the admission of the first Covid-19 patient up until October 1, 2022. Continuous variables are presented as means with standard deviation (SD), and in case of non-normal distribution as median with interquartile ranges (IQR). Categorical variables are presented as frequencies and proportions. The standardized mean difference (SMD) was used to examine balance in the pre-merger an post-merger cohorts. SMD compares the difference in means in units of the pooled standard deviation, and provides an advantage over p-values, which become statically significant even for trivial differences due to the increased power in large samples [32,33]. An SMD cut-off value of <0·1 is defined as a sign of balance between groups [37].

**Fig. 1:**
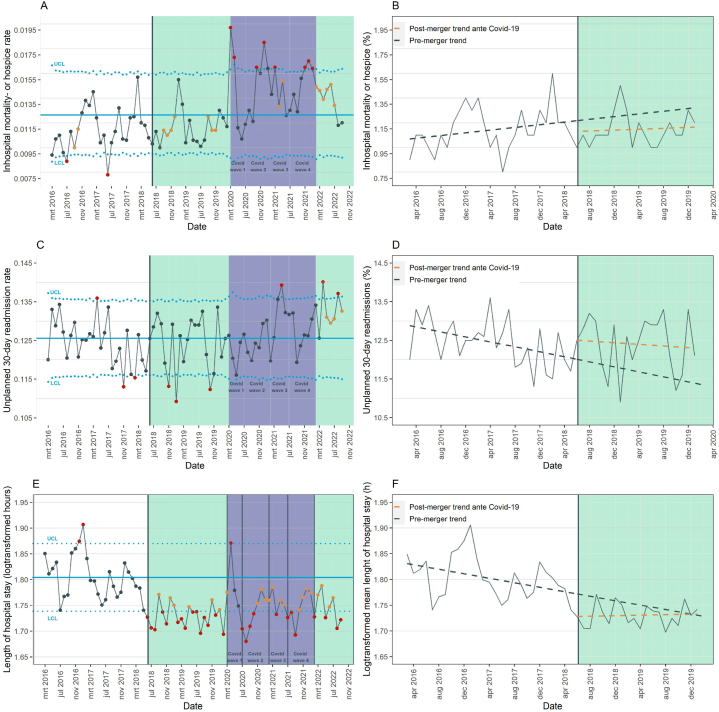
Statistical Process Control (SPC) and Interrupted Time Series (ITS) charts of crude mortality, readmissions and length of hospital stay. Panel A–B: SPC and ITS mortality, panel C–D: SPC and ITS readmissions, panel E–F: SPC and ITS length of hospital stay (log transformed mean hours). In the SPC charts, common cause variation is defined as fluctuations around the mean (solid blue line) within three SD's (dotted blue lines), with no more than six points at the same side of the mean. Special cause variation is defined as a run of more than six observations at the same side of the mean (orange points), or as any rate below or above three standard deviations (red points). The panels on the left side show SPC charts for the outcome variables including the Covid-19 period (ranging from March 2016 until the end of September 2022), the panels on the right side show ITS graphs for the period up until the outbreak of Covid-19 (ranging from March 2016 until February 2020). The purple areas indicate the consecutive Covid-19 waves in the Netherlands. The coefficients for the aggregated unadjusted ITS graphs can be found in eTable 1.

To determine potential QoC changes post-merger, complementary statistical process control (SPC) and interrupted time series (ITS) analyses were conducted for mortality, readmissions, and length of hospital stay (**eText 2**) [34]. SPC charts were also constructed for the aggregated yearly patients' hospital ratings. Yet, this aggregation level provided no sufficient data points to calculate solid pre- and post-merger trends for ITS analysis. SPC analysis' for the clinical outcome measures were performed on raw monthly data, and comprise the entire post-merger period (including Covid-19). As appropriate, P-type SPC charts were generated for mortality and readmissions, and xbar.one type SPC charts for length of hospital stay and patients' hospital rating [35]. The main ITS analysis’ were performed on patient-level data up until the admission of the first Covid-19 patient, first unadjusted and thereafter adjusted for known confounders (age, sex (as registered in EHR), hospital location, Charlson Comorbidity Index (CCI, retrospectively calculated based on ICD-10 codes), type of admission (surgical/medical), urgency of admission (elective/acute) and season of admission). For the ITS models with binary outcome variables (mortality, readmissions) logistic models were conducted. For the continuous variable, length of hospital stay, linear models were estimated, after applying a logarithmic transformation to reduce the skewed data distribution (logarithmic transformation was found to be superior to a gamma regression model [36], eFig. 2). The regression coefficients from this last model were transformed back, and converted to change percentages per month to facilitate interpretation. All ITS models included a time variable representing the pre-merger trend line, a merger variable representing any level change in the post-merger period, and an interaction term between time and merger representing the post-merger trend (**eText 2**). In all ITS models, the continuous covariate time was set to zero at the month of the merger in order to calculate the merger level-change at the intercept (**eText 2**). The null-hypothesis for the ITS models is that the pre-merger trend would have continued without the merger. To enhance data visualization, unadjusted monthly ITS coefficients (eTable 1) and graphical models (Fig. 1) are provided. Significance was defined as a two-sided p-value below 5 %.

Examining autocorrelation involved decomposition of the data to assess seasonality, and evaluation of autocorrelation function plots (ACF) (eFig. 3). Furthermore, the Durbin-Watson test was performed to check for remaining autocorrelation in the adjusted analysis’, values between 1·5-2·5 were considered as no autocorrelation (eFig. 3).

The dataset included 46 admissions with a missing discharge date, these observations were listwise deleted. More information about the coding of variables can be found in the supplementary material (**eText 2**). All analysis’ were performed in RStudio version 4.2.1 (Used packages: **eText 3**). The paper follows the Standards for Quality Improvement Reporting Excellence (SQUIRE) guideline. The Medical Ethical Review Board of Amsterdam UMC location VUmc waived the review of this study, as it falls outside the scope of the Act of Medical Research with Human Subjects (nr. 2022.0498).

## 3 Results

The mean (SD) age of the 573 813 included patient admissions was 54·3 (18·9) years, the full sample included 277 817 (48·4 %) women, 10·2 % (n = 58 436) was admitted with a surgical disease related group, and 53·8 % (n = 308 597) was admitted urgently (Table 1). Clinical characteristics of patients in the pre- and post-merger groups were found to be balanced (SMD's < 0·1), except for differences in the Charlson Comorbidity Index, and the number of admitted patients with Covid-19 in the post-merger since Covid-19 group (Covid-19 incidence Amsterdam UMC: eFig. 4).

**Table 1:** Patient characteristics at time of admission stratified to pre- and post-merger period. The pre-merger period lasted from March 2016 up until June 2018. The post-merger period was divided in an ‘ante Covid-19’ group (from June 2018 to February 2020), and a post-merger period since the admission of the first Covid-19 patient (February 2020 to September 2022).

	**Pre-merger**	**Post-merger**		**Post-merger**	
		***Ante Covid-19***		***Since Covid-19***	
**Admitted patients, No.**	**n = 202 104**	**n = 156 389**		**n = 215 320**	
**Characteristic**			**SMD**		**SMD**
Age (y) (mean (SD))	53·9 (18·8)	54·4 (18·9)	0·025	54·8 (19·0)	0·045
Women (n, %)	97 278 (48·1)	75 864 (48·5)	0·008	104 675 (48·6)	0·010
Hospital location VUmc (n, %)	101 509 (50·2)	79 132 (50·6)	0·007	112 056 (52·0)	0·036
Charlson Comorbidity Index (median [IQR])	1·0 [0·0, 3·0]	2·0 [0·0, 3·0]	0·056	2·0 [0·0, 3·0]	0·127
Surgical DRG (n, %)	21 222 (10·5)	15 934 (10·2)	0·010	21 280 (9·9)	0·020
Urgency (n, %)			0·037		0·061
Elective	90 079 (44·6)	72 616 (46·4)		102 521 (47·6)	
Acute	112 025 (55·4)	83 773 (53·6)		112 799 (52·4)	
Covid-19 (n, %)	0 (0·0)	0 (0·0)	<0·001	3907 (1·8)	0·192
ICU admission^a^ (n, %)	4434 (2·2)	3388 (2·2)	0·002	4951 (2·3)	0·007
Length of hospital stay (h) (median [IQR])	3·7 [2·0, 13·3]	3·6 [2·0, 9·8]	0·021	3·6 [2·0, 10·7]	0·017
Readmissions^b^ (n, %)	25 128 (12·4)	19 390 (12·4)	0·001	27 533 (12·8)	0·011
Mortality^c^ (n, %)	2319 (1·1)	1821 (1·2)	0·002	3106 (1·4)	0·026

Abbreviation: SD, standard deviation, SMD, standardized mean difference, IQR, interquartile range, DRG, diagnose related group, ICU, intensive care unit. a· Admission to the ICU of one of the two merged hospitals, b· Unplanned 30-day readmissions to one of the two merged hospitals, c· In-hospital mortality or discharge to hospice.

### 3.1 Mortality

The unadjusted mortality did not change for the post-merger ante Covid-19 group (Table 1), and consistently the SPC analysis shows predominantly common cause variation in the pre-merger and the post-merger ante Covid-19 period (Fig. 1). Accordingly, the adjusted ITS analysis shows no change in mortality related to the merger in this period (Table 2). Comparing pre-merger admissions to the post-merger since Covid-19 group, the unadjusted mortality increased from 1·147 % to 1·468 %, a 0·295 % absolute increase (95 % CI, 0·254 % to 0·336 %) (Table 1). This is in line with the distinct pattern of special cause variation in the direction of elevated mortality rates in the SPC graph, coinciding with the first national Covid-19 wave (Fig. 1).

**Table 2:** Patient-level interrupted time series of mortality, readmissions and length of hospital stay. Unadjusted- and adjusted patient-level interrupted time series analysis of in-hospital mortality or discharge to hospice, unplanned 30-day readmissions to one of the two merged hospitals, and length of hospital stay. The pre-merger trend is determined based on 202 104 admissions, and the post-merger period is based on 156 389 patient admissions. The pre- and post-merger trend change reflects change per month.

Outcome	Pre-merger trend		Post-merger step change		Post-merger trend	
Unadjusted	**OR (95 % CI)**	**P-value**	**OR [95 % CI]**	**P-value**	**OR (95 % CI)**	**P-value**
Mortality	1·006 (1·000 to 1·011)	0·040	0·911 (0·806 to 1·029)	0·132	0·997 (0·988 to 1·007)	0·590
Readmissions	1·005 (1·004 to 1·006)	<0·001	0·991 (0·965 to 1·018)	0·500	0·995 (0·993 to 0·997)	<0·001
	**Change (%) (95 % CI)**	**P-value**	**Change (%) (95 % CI)**	**P-value**	**Change (%) (95 % CI)**	**P-value**
Length of hospital stay (h)^b^	−0·182 (−0·281 to −0·083)	<0·001	−4·917 (−7·063 to −2·722)	<0·001	0·210 (0·029 to 0·390)	0·023
Adjusted^a^	**OR (95 % CI)**	**P-value**	**OR [95 % CI]**	**P-value**	**OR (95 % CI)**	**P-value**
Mortality	1·003 (0·997 to 1·008)	0·372	0·972 (0·855 to 1·106)	0·668	0·994 (0·984 to 1·004)	0·210
Readmissions	1·003 (1·001 to 1·004)	<0·001	0·994 (0·965 to 1·023)	0·669	0·996 (0·994 to 0·998)	<0·001
	**Change (%) (95 % CI)**	**P-value**	**Change (%) (95 % CI)**	**P-value**	**Change (%) (95 % CI)**	**P-value**
Length of hospital stay (h)^b^	−0·134 (−0·219 to −0·048)	0·002	−3·798 (−5·689 to −1·870)	<0·001	−0·155 (−0·308 to −0·001)	0·048

Abbreviation: OR, odds ratio, CI, confidence interval.

a Adjusted for age, sex, hospital, Charlson Comorbidity Index, urgency of admission, surgical vs medical and season.

b Coefficients were log-transformed before linear regression analysis, and subsequently transformed back, followed by subtracting 1, and thereafter multiplied by 100 to report change %.

### 3.2 Readmissions

Unadjusted unplanned 30-day readmission rates did not change in the post-merger ante Covid-19 period, which is consistent with the SPC chart that shows solely common cause variation. Accordingly, ITS analysis demonstrated no merger-related immediate change in readmissions post-merger. However, the adjusted ITS analysis shows a slight decrease in the post-merger readmission trend-line (OR 0·996, 95 % CI, 0·994 to 0·998; P < 0·001), representing a 0·996 odds of readmission with each extra month in the post-merger period (Table 2). The SPC graph shows that just after the last national Covid-19-wave special cause variation arises in the direction of increased readmission incidence (Fig. 1).

### 3.3 Length of hospital stay

The SPC chart for length of hospital stay demonstrates solely special cause variation, with all measurements indicating a shortened length-of-stay in the post-merger ante Covid-19 period (Fig. 1). Accordingly, the ITS analysis showed an immediately shorter length of hospital stay just after the merger (−3·798 %, 95 % CI, −5·689 % to −1·870 %; P < 0·001), and a slight but significant downward trend change in the first 20 months after the merger (−0·155 % per month, 95 % CI, −0·308 % to −0·001 %; P = 0·048 Table 2). During the first national Covid-19 wave, the SPC chart returns towards common cause variation, indicating a comparable length of stay with the pre-merger period. From the second Covid-19 wave onwards all measurements again indicate a decreased length of hospital stay.

### 3.4 Patients’ hospital rating

**Fig. 2:**
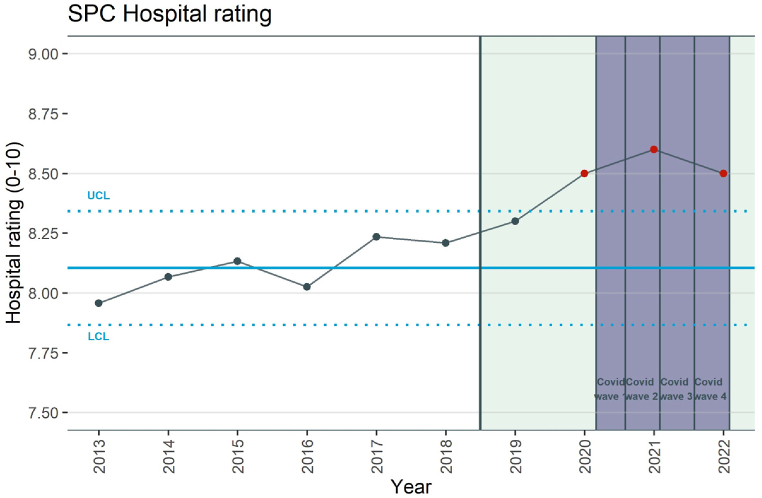
Statistical Process Control (SPC) chart of patients' hospital rating pre- and post-merger. SPC chart of average patients' hospital rating on a 1–10 scale. Common cause variation is defined as any fluctuation around the mean (solid blue line) within three SD's (dotted blue lines), with no more than six points at the same side of the mean. Special cause variation (any rating below or above three standard deviations) is indicated in red. The purple areas indicate the consecutive Covid-19 waves in the Netherlands.

The SPC chart representing patients' hospital ratings shows the average annual grades assigned by patients to the hospitals on a 1 to 10 scale (Fig. 2). Common cause variation is observed until the first post-merger year, followed by special cause variation for the years 2020, 2021 and 2022. The special cause variation indicates higher patients’ hospital ratings in the post-merger period, yet the upward trend already emerged in the pre-merger period (Fig. 2).

## 4 Discussion

This study found that the merger of two academic hospitals in the Netherlands, which implemented a comprehensive long-term integration approach, was not associated with changes in in-hospital mortality in the first 20 months after the merger. ITS analysis showed stable in-hospital mortality rates, and no immediate, nor major structural impact on unplanned 30-day readmissions. However, an immediate reduction, and a structural trend change towards decreased length of hospital stay were observed post-merger. Finally, patients’ hospital ratings seemed to improve post-merger.

A similar study on the merger of New York University Langone Hospital-Brooklyn (NYULHB) reported a transient post-merger rise in mortality rates, followed by improvements 2–3 years later [6]. One of the main differences between that case and the case evaluated in the present study are their respective merger approaches: NYULHB identified pre-merger QoC improvement opportunities, placing strong emphasis on enhancing performance in these areas through targeted initiatives, whereas the Amsterdam UMC merger employed a more comprehensive- and long-term integration strategy, emphasizing further quality improvements through academic health services concentration. This may suggest that a comprehensive long-term integration strategy prevents severe initial deterioration, but also prolongs the time needed for any potential improvements to manifest. However, another explanation for the improved post-merger mortality ratios in the NYULHB case could be a larger margin for improvement, as baseline mortality was higher [6].

Prior studies reported no merger-related changes in readmission rates post-merger, which is generally in line with the minimal changes found in the present study [1,6,37]. The apparent decrease in hospital stay duration appears noteworthy; however, from a practical standpoint, it results in a modest average reduction per admission of less than an hour. However, noteworthy is that pre-merger a minimal decrease in the length of hospital stay and a slight rise in readmission rates was seen, suggesting a trend towards improved efficiency at the expense of increased readmission rates. Post-merger, the odds of being readmitted shifted towards a decreasing trend, and there was a continuous reduction in length of hospital stay. This suggests a post-merger QoC improvement.

Contrary to this study's findings of improved patients' hospital rating post-merger, which align with the NYULHB case, most research on hospital M&A's indicates decreased or unimproved patient satisfaction post-merger [1,6,37,38]. What the NYULHB and the present case have in common is that both cases are about (an) academic hospital(s), and both cases put an emphasis on post-merger QoC improvement. These similarities may explain the positive findings. Yet, while the found rise in patient hospital rating's in this study coincides with the merger, the findings cannot conclusively be attributed to the consolidation, as an upward trend was already seen pre-merger.

The SPC analysis showed immediate increases in mortality and prolongation of the length of hospital stay coinciding with the onset of the Covid-19 pandemic. While the observed deterioration could potentially be associated with the merger – merger wave two was initiated in January 2020 – an additional contributing factor is the undeniable global impact of the Covid-19 pandemic, further underscoring the intricate interplay of multiple variables influencing healthcare outcomes during this period [39].

At last, several studies have documented a negative correlation between concentration of healthcare markets and QoC [12,13,24]. However, current dynamics in the Dutch healthcare market show waiting lists for many health services, indicating that demand for healthcare exceeds the supply [40]. In theory this gives health service providers, independent of their market position, a strong bargaining position towards health insurers, which may result in decreased incentives for QoC improvement. The merger in this study doubled the top-care market share of the hospitals to 30–40 %, yet no trend towards decreased QoC was found.

## 5 Limitations of the study

The study findings must be considered alongside several limitations. First, the study lacked a control group to correct for secular trends such as Covid-19. However, the chosen approach for the ITS has led to results that evaluate the first 20 months post-merger (ante Covid-19), and the SPC charts clearly indicate where any effects of the pandemic were potentially visible. Second, the merger was treated as a single event, whereas the long-term integration approach with successive merger waves makes the start date of the merger ambivalent. Post-merger integration processes are not yet completed and QoC changes may occur in a later stage. Third, the SPC charts for mortality, readmissions and length of hospital stay show special cause variations in the pre-intervention period, and therefore the findings should be treated with caution. However, the ITS analysis' are in line with the SPC findings. Fourth, the CQI and PEM surveys that were used to examine patients' hospital rating, had differing process- and administration methods which may have influenced the results. Finally, the clinical outcome measures may not capture quality across all medical conditions, and patients’ hospital rating is a small element of patient experience. Nevertheless, the used clinical measures are reliably and above all recognized as parameters of QoC [1,6,15,27,28].

## 6 Conclusion

This quality improvement study revealed that a full- and long-term integration strategy to a Dutch academic hospital merger was not associated with early changes in in-hospital mortality, and yielded slight improved results for secondary quality of care outcomes. The gradual integration strategy possibly helped to mitigate the adverse impact of major organizational change, but may also have postponed major positive quality of care changes. Repeated analysis’ after a longer time period could provide additional insights. A deeper understanding of facilitators of successful hospital mergers, may help to critically assess intended mergers, and provide insights in what factors can contribute to enhanced quality of care for patients.

## Funding

Amsterdam UMC institutional funding.

## Data sharing statement

With publication, the data that support the findings of this study are available for sharing. Following publication, data requests can be made to author I.J.A.W. After showing a methodologically sound proposal, compliant with our local privacy regulations, researchers can be granted access to the data. A data access agreement must be signed in advance. All code used in this study is available for sharing, requests can be made to the author I.J.A.W. (i.j.wissink@amsterdamumc.nl).

## CRediT authorship contribution statement

**Ilse J.A. Wissink:** Conceptualization, Data curation, Formal analysis, Investigation, Methodology, Project administration, Resources, Supervision, Visualization, Writing – original draft, Writing – review & editing. **Michiel Schinkel:** Conceptualization, Data curation, Formal analysis, Investigation, Methodology, Resources, Software, Supervision, Writing – review & editing. **Hessel Peters-Sengers:** Conceptualization, Data curation, Formal analysis, Investigation, Methodology, Visualization, Writing – review & editing, Validation. **Simon A. Jones:** Methodology, Validation, Writing – review & editing. **Alexander P.J. Vlaar:** Writing – review & editing. **Karen J. Kruijthof:** Writing – review & editing. **W. Joost Wiersinga:** Supervision, Visualization, Writing – review & editing.

## Declaration of generative AI and AI-assisted technologies in the writing process

During the preparation of this work the author(s) used ChatGPT in order to revise sentences. After using this tool/service, the author(s) reviewed and edited the content as needed and take(s) full responsibility for the content of the publication.

## Declaration of competing interest

The authors declare the following financial interests/personal relationships which may be considered as potential competing interests:IJA Wissink reports a relationship with Amsterdam University Medical Centres that includes: employment. APJ Vlaar reports a relationship with Amsterdam University Medical Centres that includes: employment. M Schinkel reports a relationship with Amsterdam University Medical Centres that includes: employment. K Kruijthof reports a relationship with Amsterdam University Medical Centres that includes: board membership. H Peters-Sengers reports a relationship with Amsterdam University Medical Centres that includes: employment. WJ Wiersinga reports a relationship with Amsterdam University Medical Centres that includes: employment. S. A. Jones has no competing financial interests or personal relationships that could have appeared to influence the work reported in this paper.
