# Supplementary material for: Quality of care after a horizontal merger between two large academic hospitals
Source: Heliyon. 2024 Sep 29;10(19):e38311. doi: 10.1016/j.heliyon.2024.e38311 (PMC11490856; doi:10.1016/j.heliyon.2024.e38311)
Supplement: Multimedia component 1 [file mmc1.docx]

## **Supplementary material**

**Quality of care after a horizontal merger between two large academic hospitals**

*Ilse J.A. Wissink* MD^1,2,3,7^, Michiel Schinkel PhD^1,2^, Hessel Peters-Sengers PhD ^1,2,4^, Simon A. Jones PhD ^5^, Alexander P.J. Vlaar PhD^1,3^, Karen J. Kruijthof PhD^6^, W. Joost Wiersinga, PhD^1,2^*

^1^Department of Medicine, Division of Infectious Diseases, Amsterdam UMC, University of Amsterdam, Amsterdam, The Netherlands

^2^Center for Experimental and Molecular Medicine (CEMM), Amsterdam UMC, location AMC, University of Amsterdam, Amsterdam, The Netherlands

^3^Department of Intensive Care, Amsterdam UMC, University of Amsterdam, Amsterdam, The Netherlands

^4^ Epidemiology and Data Science, Amsterdam UMC, Vrije Universiteit Amsterdam, Amsterdam, The Netherlands

^5^ Centre for Health and Delivery Science, NYU Grossman School of Medicine, New York, United States of America

^6^ Board of Directors, Amsterdam UMC, Vrije Universiteit Amsterdam, De Boelelaan 1117, Amsterdam, The Netherlands

**Table of content**

eFigure 1 - Description of the merger: processes and timeframe3

eText 1 - Dutch healthcare market and regulated competition4

eText 2 - Methods and regression models5

eFigure 2 - Histograms deviance residuals log and gamma regression models8

eTable 1 – Aggregated unadjusted monthly interrupted time series of mortality, readmissions and length of hospital stay9

eFigure 3 - Insights in autocorrelation10

eText 3 - RStudio: used packages12

eFigure 4 – Number of Covid-19 patients admitted to Amsterdam UMC 2020-202213

References14

### **eFigure 1 – Description of the merger: processes and timeframe**


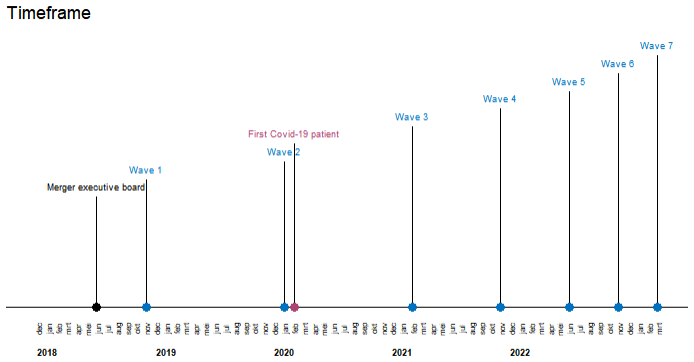


eFigure 1 - Description of Amsterdam UMC merger: processes and timeframe. The merger on the 6th of June 2018 was the start of a comprehensive and long-term integration strategy.^1,2^ One of the main objectives for the horizontal merger was to further improve the QoC through concentration of academic health services.^3-5^ The central planning, and the harmonization and- integration strategy was and is outlined by two multidisciplinary steering groups run by representatives of the executive board and senior management of clinical divisions. Simultaneous consolidation processes occur at the central and decentral level. On the decentral level, the clinical departments integrate and harmonize successively in so called ‘merger waves’. The integrating departments are supported by the two steering groups with practical matters, such as planning and risk assessments, and with the more complex human integration processes. All departments were requested to appoint one director to manage both locations, and the most departments succeeded to accomplish this within the first years after the merger. Heads of the departments were granted a high level of autonomy, but had to execute the consolidation in a budget neutral manner. Job security for medical staff was ensured for the first post-merger years. The merger started with the consolidation of the executive board on the 6^th^ of June 2018, and the subsequent merger waves were characterized as follows: wave 1: pediatric ICU, orthopedics, pulmonary surgery- and oncology, ophthalmology, dermatology; wave 2: benign gynecology, nephrology including transplantation program, clinical rheumatology, clinical pediatrics, upper-gastro-intestinal care pathway; wave 3: cardiac surgery, vascular surgery, inflammatory bowel diseases/colon, clinical pulmonary diseases, urology(adults), hepato-pancreato-billiary care pathway, medical oncology; wave 4: neonatal intensive care unit, clinical obstetrics; wave 5: outpatient clinic obstetrics, clinical obstetrics; wave 6: oral- and maxillofacial surgery, ear-nose-throat surgery, mamma care, outpatient clinic movement disorders, elective neurosurgery; wave 7: trauma care and acute neurology. On the central hospital level the human resources, information technology and facility management departments have been integrated in the first post-merger years, even as the department responsible for risk monitoring and quality of care. The same electronic health records systems were already adopted in 2016. Moreover, the scientific departments of both hospitals have been consolidated into one doctoral school, and also the postgraduate medical training programs have been merged. The affiliated medical schools did not merge. The harmonization and integration practices are expected to continue until at least 2025.

## **eText 1 - Dutch healthcare market and regulated competition**

In 2006 market-based principles have been introduced to the Dutch healthcare system in the form of a regulated healthcare market.^6^ In this system all Dutch citizens are obliged to buy basic health insurance from a private health insurer which covers costs for essential healthcare such as general practitioners and hospitals within the contracted network of the insurer.^6^ Before healthcare expenses are fully reimbursed, policyholders are subject to an annual deductible. Enrollees can choose their preferred deductible, which for example in 2023 ranged from €385 to €885, where higher deductibles result in discounted monthly premiums.^7^
 Health insurers are obliged to accept each enrollee and have the responsibility to guarantee healthcare services for their policyholders. Consequently, health insurers engage in yearly negotiations with health service providers to make agreements on healthcare prices and quality. Approximately 70% of medical specialist healthcare is freely negotiable.^8^
 Nationally, the merger has reduced the number of academic hospital organizations from eight to seven. The antitrust authority refrained from assessing relative market power in relation to the other academic hospitals, citing that a small proportion of ‘top care’ - care provided to patients with complex care requirements and or more serious illnesses - is of such specialized nature that competitive dynamics are not applicable.^1^ The potential impact of the merging entity on market power in its geographic region was assessed (region North-Holland+), and multiple hospitals in the same region were considered competitors leading to an estimated pre-merger market share of 10-20% for both hospitals for the ‘top-care’-segment. The merger was expected to increase the market share in this segment to approximately 30-40%.^1^ The merger was approved by the Dutch antitrust authority (ACM) in September 2017.^1^

## **eText 2 – Methods and regression models**

SPC is a statistical technique that uses time-series data to visually display control charts, and allows for identification of random fluctuations (‘common cause variation’) when the outcome variable is in control, or ‘special cause variation’ when not in control.^9^ ITS analysis is a statistical method that quantifies changes in level and trend from before to after an interruption (merger). ITS assumes that the observations from the pre-merger period predict where future observations would lie in the absence of the merger, and can be added to SPC analysis since it does not require a stable baseline and provides the opportunity to correct for autocorrelation.^9^
 All ITS models in the present study included a time variable representing the pre-merger trend line, a merger variable representing any level change in the post-merger period, and an interaction term between time and merger representing the post-merger trend.

Figure 1 in the main article shows on the right side ITS graphs of the unadjusted monthly aggregated coefficients (most basic model). The level- and step change coefficients from these models can be found in eTable1. The applied monthly aggregated unadjusted regression models are outlined here:

- Ordinary Least Squares (OLS) model for unadjusted monthly aggregated mortality or hospice:

ols(Inhospital_mortality_mean ~ time2 + merger + time2*merger, data=time_series)

- OLS model for monthly unadjusted unplanned 30-day-same-hospital-readmissions:

ols(Unplanned_30day_readmission_mean ~ time2 + merger + time2*merger, data=time_series2)

- OLS model for monthly unadjusted length of hospital stay:

ols(Duration_of_admission_log_mean ~ time2 + merger + time2*merger, data=time_series3)

Table 2 in the main article shows the unadjusted and adjusted patient level coefficients of the ITS analysis (and therefore deviate somewhat from the coefficients shown in the ITS graphs in figure 1). The applied patient level unadjusted ITS models for the upper part of table 2 are outlined here:

- Logistic regression model for unadjusted patient-level in-hospital mortality or hospice:

lrm(Died_inhosp_or_hospice ~ time2 + merger + time2*merger, data = df10_new)

- Logistic regression model for unadjusted patient-level unplanned-30-day-same-hospital-readmissions:

lrm(readmission_30 ~ time2 + merger + time2*merger, data = df10_new)

- OLS model for unadjusted patient-level length of hospital stay:

ols(duration_admission_hours_log ~ time2 + merger + time2*merger, data = df10_new)

The applied patient level adjusted ITS models for the lower half of table 2 are outlined here:

- Logistic regression model for adjusted patient-level in-hospital mortality or hospice:

lrm(Died_inhosp_or_hospice ~ time2 + merger + time2*merger + Season + Age + Sex + Hospital + Charlson + Surgery_medicine + Urgency_of_admission2, data = df10_new)

- Logistic regression model for adjusted patient-level unplanned-30-day-same-hospital-readmissions:

lrm(readmission_30 ~ time2 + merger + time2*merger + Season + Age + Sex + Hospital + Charlson + Surgery_medicine + Urgency_of_admission2, data = df10_new)

- Linear regression model for adjusted patient-level length of hospital stay:

ols(duration_admission_hours_log ~ time2 + merger + time2*merger + Season + Age + Sex + Hospital + Charlson + Surgery_medicine + Urgency_of_admission2, data = df10_new)

To determine the level-change at the time of the merger (b2 in figure below), the continuous covariate time was transformed as a negative variable in the pre-merger period and as a positive variable in the post-merger period. In this way, time was set to zero at the month of the merger. Looking at the formula Y = b0 + time*b1 + merger*b2 + time*merger*b3, it becomes clear that at time = 0, the formula indicates the level-change at the time of the merger. Illustrated graphically:


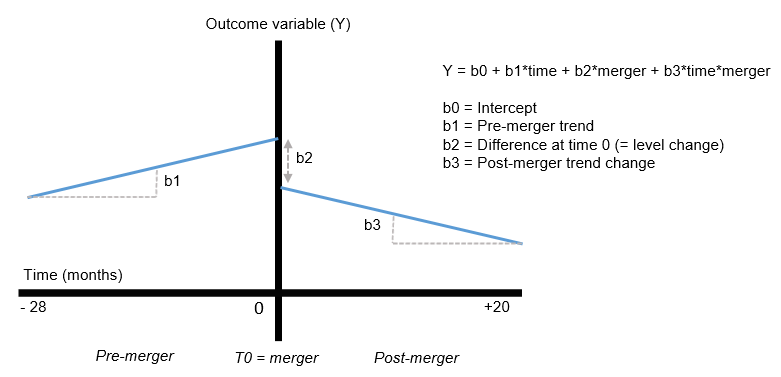


**Coding of variables**

Few small categories in the urgency-of-admission variable (total n = 544) were added to the most logical group (either elective or acute). For three admissions, the ICU-admission variable had an atypical value and was adjusted to indicate a non-ICU-admission.

**eFigure 2 – Histograms deviance residuals log and gamma regression models**


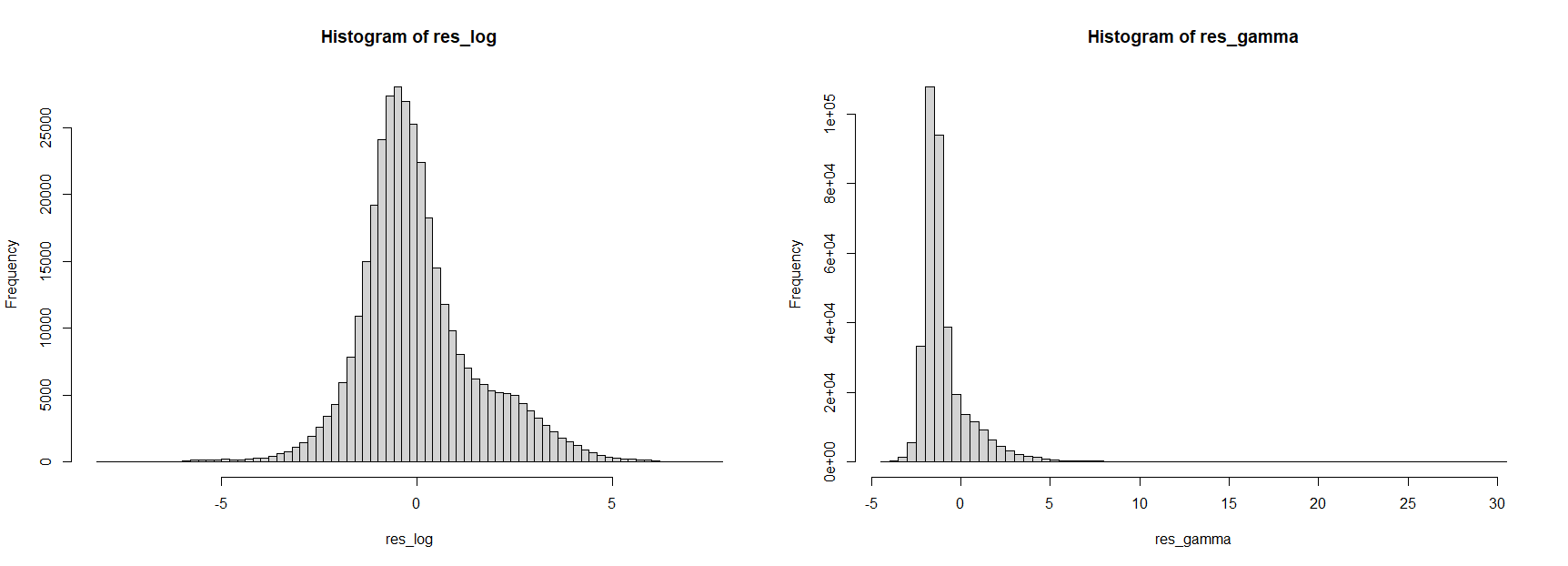


The length of hospital stay data was found to be non-normally distributed. This figure shows histograms of the deviance residuals of the log model (left) and gamma model (right). Based on these histograms, de log transformed model for length of hospital stay was found to be superior to a gamma regression model.

**eTable 1 - Aggregated unadjusted monthly interrupted time series of mortality, readmissions and length of hospital stay**

Aggregated unadjusted monthly interrupted time series analysis of in-hospital mortality or discharge to hospice, unplanned 30-day readmissions to one of the two merged hospitals, and length of hospital stay. The pre-merger trend is determined based on 202 104 patient admissions, and the post-merger period is based on 156 389 admissions. The pre- and post-merger trend change reflects change per month.

| **Outcome** | **Pre-merger trend** |  | **Post-merger**  **level change** |  | **Post-merger**  **trend change** |  |
| --- | --- | --- | --- | --- | --- | --- |
| Unadjusted | **Change (%) (95% CI)** | **P-value** | **Change (%) (95% CI)** | **P-value** | **Change (%) (95% CI)** | **P-value** |
| Mortality | 0·007 (-0·000 to 0·015) | 0·063 | -0·144 (-0·321 to 0·033) | 0·117 | -0·004 (-0·018 to 0·010) | 0·608 |
| Readmissions | -0·037 (-0·067 to  -0·006) | 0·025 | 0·576 (-0·155 to 1·306) | 0·130 | 0·026 (-0·032 to 0·083) | 0·387 |
| Length of hospital stay (h)* | -0·180 (-0·344 to  -0·015) | 0·038 | -4·971 (-8·603 to -1·194) | 0·014 | 0·205 (-0·102 to 0·512) | 0·197 |
|  | | | | | | |
| Abbreviation: CI, confidence interval * Coefficients were log-transformed before linear regression analysis, and subsequently transformed back, followed by subtracting 1, and thereafter multiplied by 100 to report change %. | | | | | | |

## **eFigure 3 – Insights in autocorrelation**


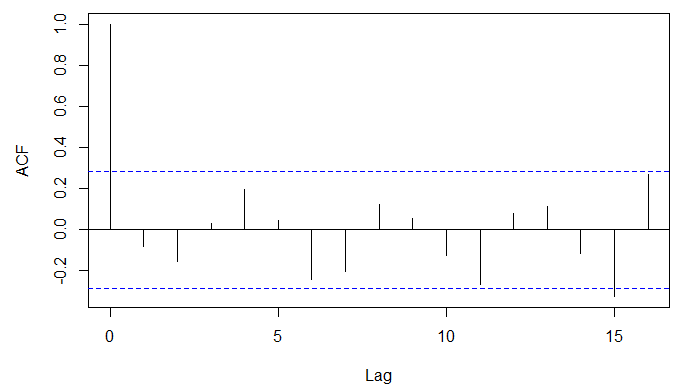

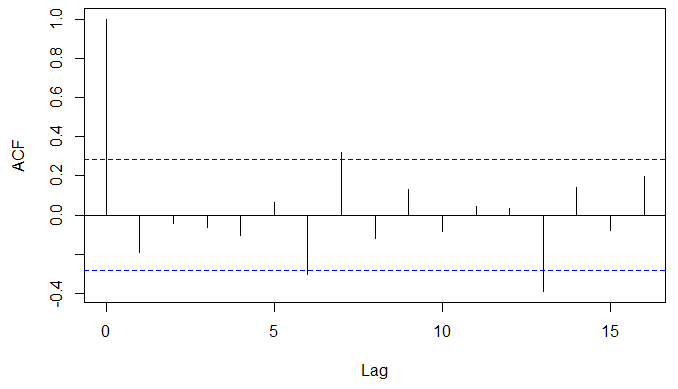
**A B**
Durbin-Watson statistic adjusted monthly model: 2·3763, p-value = 0·6297 Durbin-Watson statistic adjusted monthly model: 2·1582, p-value = 0·3317

Durbin-Watson statistic patient level adjusted model: 1·9964, p-value = 0·1381 Durbin-Watson statistic patient level adjusted model: 1·8707, p-value < 2·2e-16


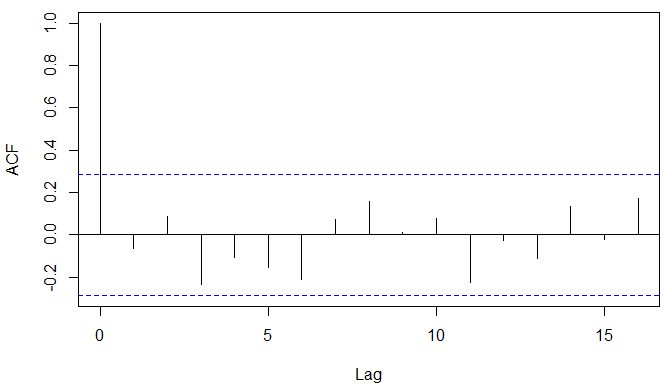
**C**

Durbin-Watson statistic adjusted monthly model: 2·1257, p-value = 0·292

Durbin-Watson statistic patient level adjusted model: 1·9345, p-value < 2·2e-16

Examining autocorrelation involved evaluation of autocorrelation function plots (ACF) with residuals of the adjusted monthly models. Potential remaining autocorrelation was checked for with the Durbin-Watson statistic of the adjusted monthly models and patient-level adjusted models. The different panels A-C show respectively in-hospital mortality or hospice, 30-day same hospital readmissions, and length of hospital stay. Durbin-Watson statistic values between 1·5-2·5 were considered as no autocorrelation.

## **eText 3 - RStudio, used packages**

## RStudio version 4.2.1

**General**

- readxl ([Read Excel Files • readxl (tidyverse.org)](https://readxl.tidyverse.org/))
- tools ([Rtools42 for Windows (r-project.org)](https://cran.r-project.org/bin/windows/Rtools/rtools42/rtools.html))
- dplyr ([r-project.org/nosvn/pandoc/dplyr.html](https://www.r-project.org/nosvn/pandoc/dplyr.html))
- tidyverse ([tidyverse: Easily Install and Load the 'Tidyverse' (r-project.org)](https://cran.r-project.org/web/packages/tidyverse/tidyverse.pdf))
- ggplot2 ([CRAN - Package ggplot2 (r-project.org)](https://cran.r-project.org/web/packages/ggplot2/index.html))
- lubridate ([CRAN - Package lubridate (r-project.org)](https://cran.r-project.org/web/packages/lubridate/index.html))

**Descriptive statistics**

- tableone ([Introduction to tableone (r-project.org)](https://cran.r-project.org/web/packages/tableone/vignettes/introduction.html))

**SPC and ITS Analysis**

- qcc ([A quick tour of qcc (r-project.org)](https://cran.r-project.org/web/packages/qcc/vignettes/qcc_a_quick_tour.html))
- rms ([CRAN - Package rms (r-project.org)](https://cran.r-project.org/web/packages/rms/index.html))
- lmtest ([lmtest: Testing Linear Regression Models (r-project.org)](https://cran.r-project.org/web/packages/lmtest/lmtest.pdf))
- stats ([R: The R Stats Package (ethz.ch)](https://stat.ethz.ch/R-manual/R-devel/library/stats/html/stats-package.html))

## **eFigure 4 – Number of Covid-19 patients admitted to Amsterdam UMC 2020-2022**


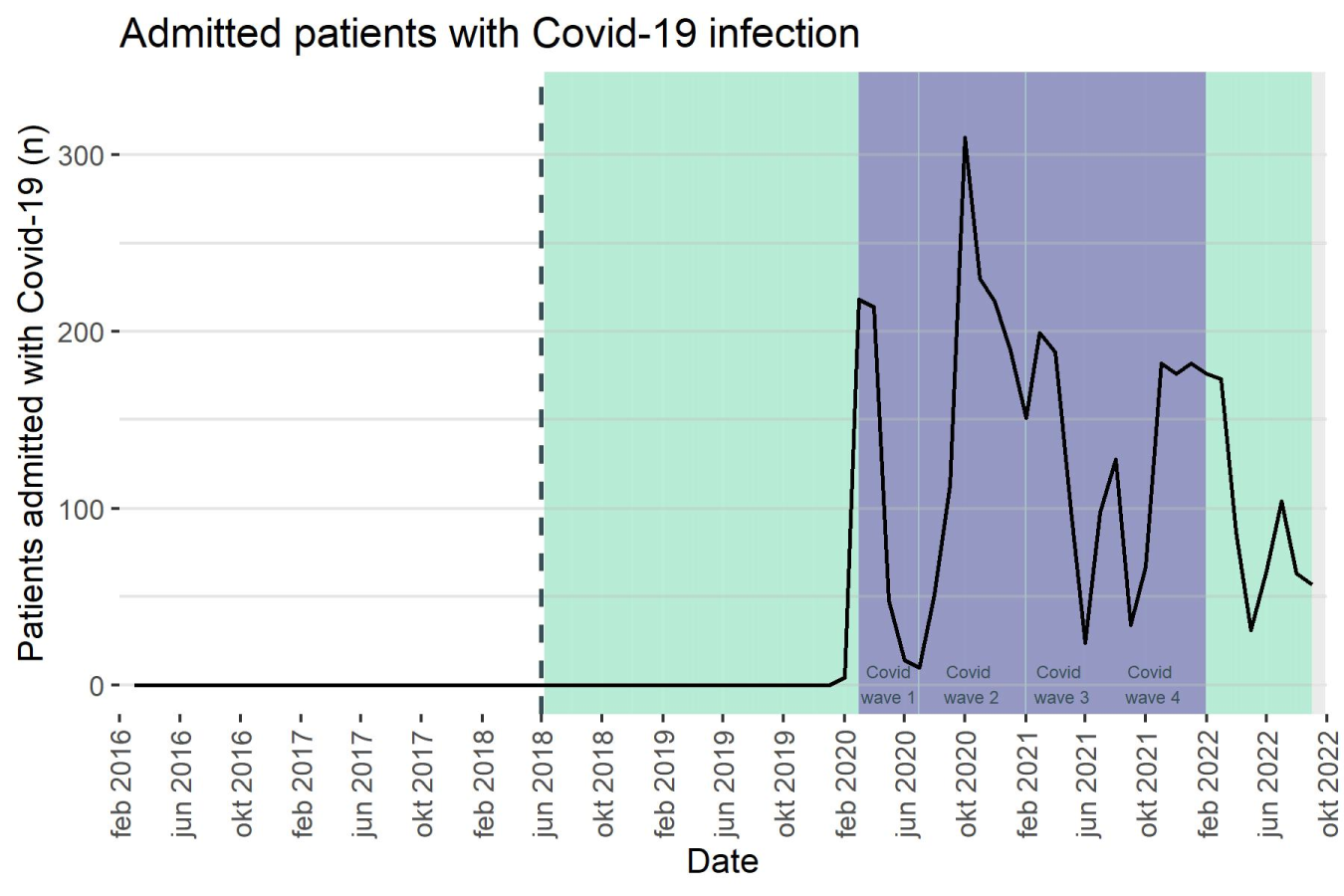


Absolute numbers of patients admitted to Amsterdam UMC with a Covid-19 infection. In the period before and after the national Covid-19 waves, the hospital treated Covid-19 patients as well.^10^ During the pandemic multiple healthcare services were scaled down which significantly changed the healthcare landscape.^11^

## **References**

1. Dutch Authority for Consumers and Markets (ACM). Granting of permit for concentration between the Academic Medical Center and the VUmc foundation: <https://www.acm.nl/sites/default/files/documents/2017-09/amc-en-vumc-krijgen-vergunning-voor-fusie-21-09-2017.pdf>. 2017. Accessed May 12, 2023.

2. Amsterdam UMC. Website publication about merger. 2018. <https://www.amsterdamumc.org/nl/organisatie/fusie.htm#:~:text=Het%20Academisch%20Medisch%20Centrum%20(AMC,een%20gezamenlijke%20naam%3A%20Amsterdam%20UMC>. (accessed 31-10-2023).

3. Academic Medical Center (AMC) and Free University Medical Center (VUmc). *Alliance AMC-V4Umc, declaration of intent and vision document*. 2011. Accessed Jan 2023.

4. Academic Medical Center (AMC) and Free University Medical Center (VUmc). *Towards an AMC-VUmc alliance.* 2013. Accessed Jan 2023.

5. Academic Medical Center (AMC) and Free University Medical Center (VUmc). *Towards excellence: ambitions, locations and plans*. 2014. Accessed Oct 2023.

6. Enthoven AC, van de Ven WPMM. Going Dutch - Managed competition health insurance in the Netherlands. *N Engl J Med* 2007; **357**(24): 2421-3.

7. Central Dutch Government. *Health insurance - Deductible - Questions and Answers:* <https://www.rijksoverheid.nl/onderwerpen/zorgverzekering/vraag-en-antwoord/eigen-risico-zorgverzekering#:~:text=U%20betaalt%20een%20eigen%20risico,zelf%20de%20eerste%20%E2%82%AC%20385>. Accessed Sep 20, 2023.

8. Westra D, Angeli F, Kemp R, Batterink M, Reitsma J. If you say so: A mixed-method study of hospital mergers and quality of care. *Health Care Manag Rev.* 2022; **47**(1): 37-48.

9. Fretheim A, Tomic O. Statistical process control and interrupted time series: a golden opportunity for impact evaluation in quality improvement. *BMJ Qual Saf* 2015; **24**(12): 748-52.

10. Slim MA, Appelman B, Peters-Sengers H, et al. Real-world evidence of the effects of novel treatments for Covid-19 on mortality: a nationwide comparative cohort study of hospitalized patients in the first, second, third, and fourth waves in the Netherlands. *Open Forum Infect Dis.* 2022; **9**(12): 1-12.

11. Dutch National Institute for Public Health and the Environment (RIVM). *Impact of the first COVID-19 wave on regular healthcare and health, inventory of the scale of the problem and a first estimate of health effects:* <https://www.rivm.nl/bibliotheek/rapporten/2020-0183.pdf>*.* 2020:156. Accessed Nov 07, 2023.
